# Glucagon-Like Peptide-1 Receptor Agonists for Treatment of Nonalcoholic Fatty Liver Disease and Nonalcoholic Steatohepatitis: An Updated Meta-Analysis of Randomized Controlled Trials

**DOI:** 10.3390/metabo11020073

**Published:** 2021-01-27

**Authors:** Alessandro Mantovani, Graziana Petracca, Giorgia Beatrice, Alessandro Csermely, Amedeo Lonardo, Giovanni Targher

**Affiliations:** 1 Section of Endocrinology, Diabetes and Metabolism, Department of Medicine, University of Verona, 37126 Verona, Italy; alessandro.mantovani@univr.it (A.M.); grazianapetracca1@gmail.com (G.P.); giorgiabeatricejb@gmail.com (G.B.); csermelyale@gmail.com (A.C.); 2 Internal Medicine, Ospedale Civile di Baggiovara, Azienda Ospedaliero-Universitaria, 41126 Modena, Italy; a.lonardo@libero.it

**Keywords:** GLP-1 receptor agonists, exenatide, liraglutide, semaglutide, dulaglutide, nonalcoholic fatty liver disease, NAFLD, nonalcoholic steatohepatitis, NASH, type 2 diabetes mellitus

## Abstract

To assess the efficacy of glucagon-like peptide-1 receptor agonists (GLP-1 RAs) for treatment of nonalcoholic fatty liver disease (NAFLD) or steatohepatitis (NASH), we performed a systematic review and meta-analysis of randomized controlled trials (RCTs). Three large electronic databases were systematically searched (up to 15 December 2020) to identify placebo-controlled or active-controlled RCTs using different GLP-1 RAs. We included eleven placebo-controlled or active-controlled phase-2 RCTs (involving a total of 936 middle-aged individuals) that used liraglutide (*n* = 6 RCTs), exenatide (*n* = 3 RCTs), dulaglutide (*n* = 1 RCT) or semaglutide (*n* = 1 RCT) to specifically treat NAFLD or NASH, detected by liver biopsy (*n* = 2 RCTs) or imaging techniques (*n* = 9 RCTs). Compared to placebo or reference therapy, treatment with GLP-1 RAs for a median of 26 weeks was associated with significant reductions in the absolute percentage of liver fat content on magnetic resonance-based techniques (pooled weighted mean difference: −3.92%, 95% confidence intervals (CI) −6.27% to −1.56%) and serum liver enzyme levels, as well as with greater histological resolution of NASH without worsening of liver fibrosis (pooled random-effects odds ratio 4.06, 95% CI 2.52–6.55; for liraglutide and semaglutide only). In conclusion, treatment with GLP-1 RAs (mostly liraglutide and semaglutide) is a promising treatment option for NAFLD or NASH that warrants further investigation.

## 1. Introduction

Nonalcoholic fatty liver disease (NAFLD) is a metabolic liver disease that encompasses a spectrum of progressive pathological conditions, ranging from simple steatosis to nonalcoholic steatohepatitis (NASH), cirrhosis and hepatocellular carcinoma (HCC) [1,2,3,4]. The incidence of NAFLD is rapidly increasing worldwide in parallel to the obesity and type 2 diabetes mellitus (T2DM) epidemics. It has been estimated that NAFLD affects up to ~30% of the general population and up to ~70–80% of people with either T2DM or obesity [5,6,7]. Strong evidence indicates that T2DM is one of the most important risk factors for faster progression of NAFLD to NASH, cirrhosis and HCC [8,9,10]. Over the last decade, it has become increasingly clear that NAFLD, especially NASH with advanced stages of fibrosis, also increases the risk of chronic vascular complications of diabetes [11]. Therefore, early recognition of NAFLD and monitoring of NASH with advanced fibrosis in people with T2DM are crucial [12,13].

NAFLD and NASH are mutually and bi-directionally associated with insulin resistance [4,8,14]. Insulin resistance is one of the key components of the metabolic syndrome and an established risk factor for the development of T2DM [14]. The link between insulin resistance and risk of T2DM involves multiple metabolic intermediates that include branched chain and aromatic amino acids, sugars and glycolysis intermediates, as well as ceramides and short-, medium- and long-chain acylcarnitines [15,16,17].

Currently, other than difficult-to-maintain lifestyle changes, there are no approved pharmacotherapies for the treatment of NAFLD or NASH. Several drug molecules with different mechanisms of action are under development to treat this common and burdensome liver disease, although their efficacy has been limited [18]. A recent Bayesian network meta-analysis of randomized controlled trials (RCTs) and non-randomized intervention studies of individuals with biopsy-proven NASH showed that pioglitazone and bariatric surgery are the two most effective treatment options for NASH, thereby supporting the notion that weight loss and improvement in hepatic insulin resistance are promising approaches for the treatment of this metabolic liver disease [19]. However, although it has been demonstrated that long-term use of pioglitazone in adults with biopsy-proven NASH has beneficial effects on the histological resolution of NASH, this drug may have some side-effects, such as moderate weight gain, peripheral oedema and risk of distal bone fractures (especially in postmenopausal women) [12,20,21].

Glucagon-like peptide-1 receptor agonists (GLP-1 RAs) are a class of subcutaneous glucose-lowering drugs approved for the treatment of T2DM [22]. Large RCTs on GLP-1 RAs have also consistently demonstrated that these drugs exert beneficial effects on the risk of adverse cardiovascular outcomes, all-cause mortality and worsening of nephropathy in patients with T2DM [22,23,24]. GLP-1 RAs improve glycemic control while also reducing body weight and insulin resistance [22]. As discussed in detail below, a number of RCTs have recently assessed the possible beneficial hepatic effects of liraglutide and other long-acting injectable GLP-1 RAs amongst adults with NAFLD, irrespective of diabetes status.

On these grounds, our systematic review and meta-analysis aimed at examining the published data of placebo-controlled or active-controlled RCTs, which tested the efficacy and safety of GLP-1 RAs to specifically treat NAFLD or NASH in adults with or without pre-existing T2DM.

## 2. Results

Supplementary Figure S1 shows the results of the literature research and study selection. We initially identified 15 potentially relevant RCTs from PubMed, Scopus and ClinicalTrials.Gov databases until to 15 December 2020 [25,26,27,28,29,30,31,32,33,34,35,36,37,38,39]. After examining the full text of these 15 articles, we excluded four studies [35,36,37,38] because of unsatisfactory inclusion criteria or unsatisfactory outcome measures, as specified in the Preferred Reporting Items for Systematic Reviews and Meta-Analyses (PRISMA) flow diagram. Therefore, a total of eleven RCTs (six placebo-controlled and five active-controlled studies) were considered eligible for inclusion in the meta-analysis and were assessed for quality.

The main characteristics of these RCTs are shown in Supplementary Table S1. In total, there were 935 middle-aged overweight or obese individuals with NAFLD or NASH (51% men; mean ± SD: age 49 ± 5 years; body mass index: 32 ± 3 kg/m^2^; serum aspartate aminotransferase (AST): 46 ± 28 UI/L; serum alanine aminotransferase (ALT): 62 ± 42 UI/L; proportion of known T2DM: 72.4%, and hemoglobin A1c level: 8.1 ± 0.6%), who were treated for a median of 26 weeks (inter-quartile range: 24–27 weeks). Of these individuals, 406 were randomly assigned to placebo or reference therapy, whereas 529 were randomly assigned to liraglutide (*n* = 6 RCTs), exenatide (*n* = 3 RCTs), dulaglutide (*n* = 1 RCT) or semaglutide (*n* = 1 RCT) to specifically treat NAFLD or NASH. The diagnosis of NAFLD was based on liver biopsy in two RCTs that included individuals with biopsy-proven NASH and varying amounts of liver fibrosis; liver ultrasonography in two RCTs, and magnetic resonance-based techniques (i.e., magnetic resonance imaging-proton density fat fraction (MRI-PDFF) or magnetic resonance spectroscopy (MRS)) in the remaining seven RCTs, respectively. The large majority of these RCTs were undertaken in people with established T2DM (*n* = 7 studies), two RCTs were conducted in individuals with and without T2DM, whereas two RCTs were conducted in non-diabetic individuals, i.e., women with polycystic ovary syndrome or non-diabetic individuals with obesity. One RCT included a multinational cohort of 320 individuals (recruited in 16 different countries), four RCTs were conducted in China, one in Singapore, one in India and four in the Europe (i.e., United Kingdom, France, Denmark, and the Netherlands). Among the eligible RCTs with available data on adverse effects, GLP-1 RAs were generally well tolerated and had a similar adverse event profile to either placebo or reference therapy, except for an increased frequency of gastro-intestinal symptoms, such as nausea, constipation, diarrhoea or abdominal discomfort. However, these gastro-intestinal symptoms were mainly transient and mild-to-moderate in severity across the included RCTs.

In Supplementary Table S2 the risk of bias for each RCT assessed by the Cochrane Collaboration’s tool is shown, which includes seven potential sources of bias. For each domain, we categorized each RCT into three categories: low, unclear, or high risk of bias.

**Figure 1:**
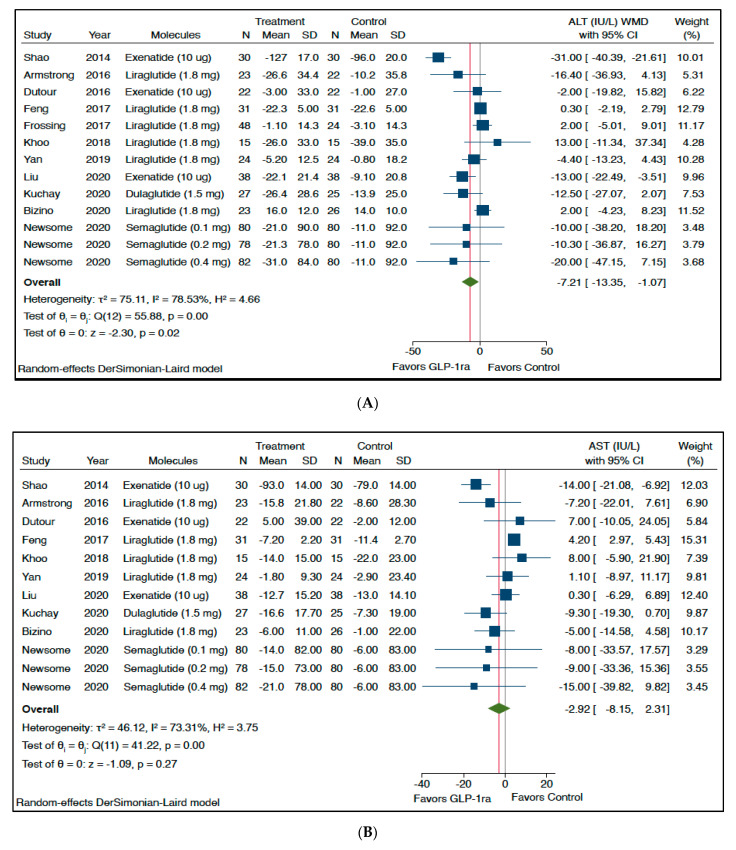

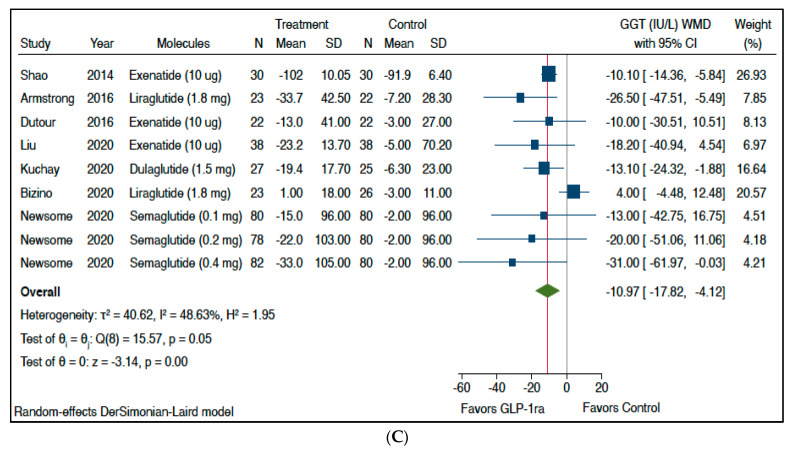
Forest plot and pooled estimates of the effects of different glucagon-like peptide-1 receptor agonists (GLP-1 Ras) on serum liver enzyme levels (i.e., serum alanine aminotransferase (ALT) (*n* = 11 RCTs included, panel (**A**)), aspartate aminotransferase (AST) (*n* = 10 RCTs included, panel (**B**)), and gamma-glutamyltransferase (GGT) (*n* = 7 RCTs included, panel (**C**))) as compared with placebo or reference therapy. The pooled (green diamond) and individual effect sizes for all RCTs included were expressed as weighted mean difference (WMD) and 95% confidence intervals (CI).

Figure 1 shows the forest plot and pooled estimates of the effect of different GLP-1 RAs on circulating levels of serum liver enzymes. When compared to placebo or reference therapy, treatment with GLP-1 RAs was associated with significant reductions in the circulating levels of serum ALT (panel A: *n* = 11 RCTs; pooled weighted mean difference (WMD): −7.21 IU/L, 95% CI −13.35 to −1.07 IU/L; *Z*-test = −2.30, *p* = 0.02) and gamma-glutamyltransferase (GGT) (panel C: *n* = 7 RCTs; pooled WMD: −10.97 IU/L, 95% CI −17.82 to −4.12 IU/L; *Z*-test = −3.14, *p* < 0.001). Serum AST levels did not differ between the two arms of treatment (panel B: *n* = 10 RCTs; pooled WMD: −2.92 IU/L, 95%CI −8.15 to 2.31 IU/L; *Z*-test = −1.09, *p* = 0.27).

**Figure 2:**
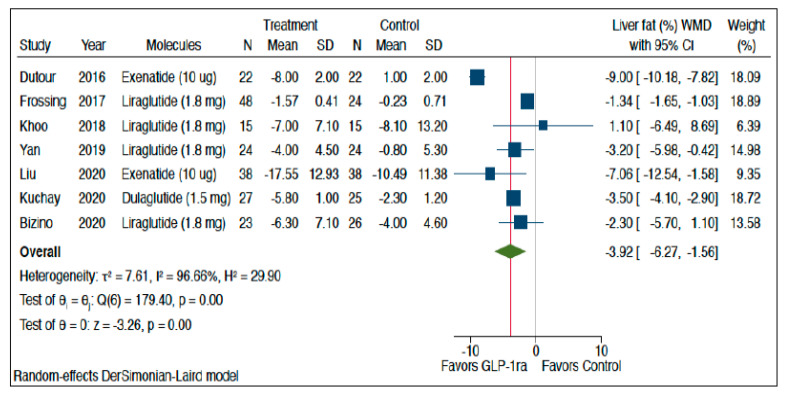
Forest plot and pooled estimates of the effect of different GLP-1 RAs on the absolute percentage of liver fat content as assessed by magnetic resonance-based techniques (*n* = 7 RCTs included) as compared with placebo or reference therapy. The pooled (green diamond) and individual effect sizes for all RCTs included were expressed as weighted mean difference (WMD) and 95% confidence intervals (CI). The blue square in the figure represents the WMD for each single RCT.

Figure 2 shows the forest plot and pooled estimates of the effect of different GLP-1 RAs on liver fat content as assessed by magnetic resonance-based techniques. Overall, in the seven RCTs included in this analysis, the pooled mean relative percent changes of liver fat content—as assessed by either MRI-PDFF or MRS among patients treated with GLP-1 RAs and those treated with placebo or reference therapy—at the end of the trials were −32% vs. −14%, respectively. As shown in the figure, when compared to placebo or reference therapy, treatment with GLP-1 RAs was associated with a significant improvement in the absolute percentage of liver fat content (*n* = 7 RCTs; pooled WMD: −3.92%, 95% CI −6.27% to −1.56%; *Z*-test = −3.26, *p* < 0.0001). A significant improvement in hepatic steatosis was also observed in the only two small RCTs conducted in China that used liver ultrasonography, but we did not perform a formal meta-analysis for these two trials.

We tested for the possibility of excessive influence of individual RCTs using an influence test that eliminated each of the included RCTs one at a time. Notably, eliminating each of the eligible RCTs from the pooled primary analysis, reported in Figure 2, did not show any effect on the observed significant improvements in liver fat content induced by GLP-1 RAs (data not shown).

**Figure 3:**
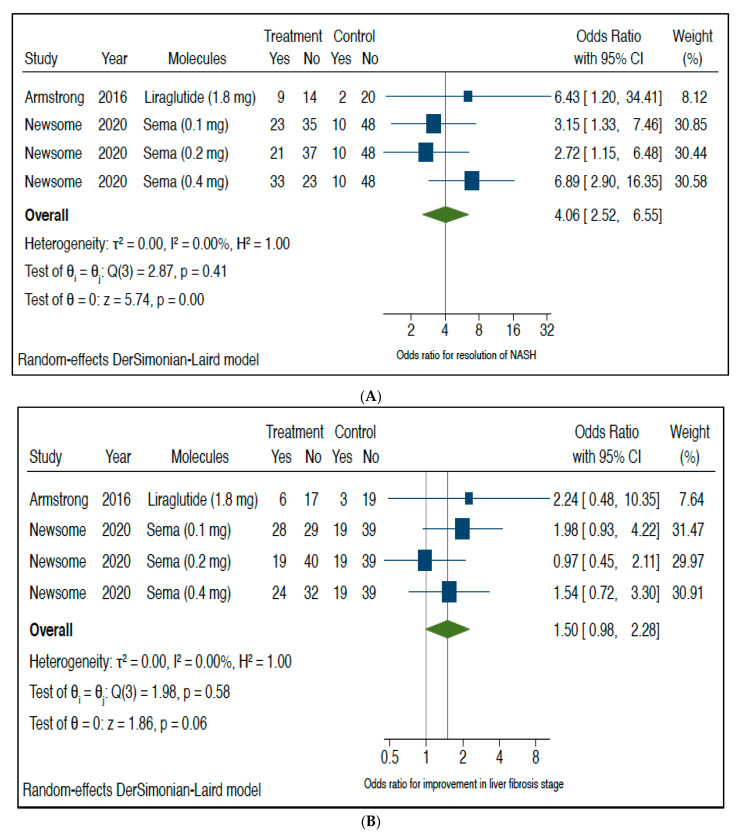
Forest plot and pooled estimates of the effect of GLP-1 RAs (*n* = 2 RCTs included using either liraglutide 1.8 mg/day or semaglutide at a dose of 0.1 mg, 0.2 mg or 0.4 mg/day subcutaneously) on histologic resolution of NASH with no worsening of liver fibrosis (panel (**A**)), and improvement in liver fibrosis stage without worsening of NASH (panel (**B**)) as compared with placebo. The pooled (green diamond) and individual effect sizes for all RCTs included were expressed as random-effect odds ratio (OR) and 95% confidence intervals (CI).

Figure 3 shows the forest plot and pooled estimates of the effect of GLP-1 RAs (*n* = 2 placebo-controlled RCTs included using either liraglutide 1.8 mg/day or semaglutide at a dose of 0.1 mg, 0.2 mg or 0.4 mg/day subcutaneously) on histologic resolution of NASH with no worsening of liver fibrosis (panel A), and improvement in liver fibrosis stage without worsening of NASH (panel B). Notably, treatment with once-daily liraglutide or semaglutide was associated with a significantly greater histologic resolution of NASH with no worsening of liver fibrosis (pooled random-effects odds ratio 4.06, 95% CI 2.52–6.55; *Z*-test = 5.74, *p* < 0.0001; I^2^ = 0%). In contrast, no significant differences were observed between the GLP-1-RA group and the placebo group in the percentage of patients who had an improvement in liver fibrosis stage without worsening of NASH (pooled random-effects odds ratio 1.50, 95% CI 0.98–2.28; *Z*-test = 1.86, *p* = 0.06; I^2^ = 0%).

As shown in Supplementary Figure S2, when compared to placebo or reference therapy, treatment with GLP-1 RAs was associated with significant reductions in body weight (panel A: *n* = 11 RCTs; pooled WMD: −4.06 kg, 95% CI −5.44 to −2.68 kg; *Z*-test = −5.76, *p* <0.0001) and hemoglobin A1c levels (panel B: *n* = 9 RCTs; pooled WMD: −0.45%, 95% CI −0.79 to −0.12; Z-test = −2.65, *p* = 0.01).

We also performed some univariable meta-regression analyses to examine the effect of potential moderator variables on the observed changes in liver fat content induced by GLP-1 RAs (Supplementary Figure S3). These analyses do not show any significant effects of age, sex or body mass index at baseline on the drug-induced improvement in the absolute percentage of liver fat content as assessed by MRI-PDFF or MRS.

As reported in Supplementary Figure S4, the Egger’s regression test did not show any statistically significant asymmetry of the funnel plots of the eligible RCTs examining the effect of GLP-1 RAs on liver fat content as assessed by magnetic resonance-based techniques (*p* = 0.701), as well as the circulating levels of serum ALT (*p* = 0.484), AST (*p* = 0.409) and GGT (*p* = 0.097), thereby suggesting that publication bias was unlikely.

## 3. Discussion

To our knowledge, compared to recently published systematic review articles on this topic [20,36,40,41], this is the largest and most updated systematic review and meta-analysis of RCTs that used different GLP-1 RAs (also including two newer long-acting injectable GLP-1 RAs, such as dulaglutide and semaglutide), for the treatment of NAFLD or NASH, irrespective of T2DM status.

The present meta-analysis includes eleven RCTs (six placebo-controlled and five active-controlled studies) testing the efficacy and safety of liraglutide (*n* = 6 RCTs), exenatide (*n* = 3 RCTs), dulaglutide (*n* = 1 RCT) or semaglutide (*n* = 1 RCT) to specifically treat NAFLD or NASH. These RCTs were conducted for a median period of 26 weeks and provide aggregate data on 935 middle-aged overweight or obese individuals for whom the diagnosis of NAFLD was based on imaging techniques (mostly magnetic resonance-based techniques), and NASH assessed histologically. The majority of the individuals included in these eleven RCTs had pre-existing T2DM (~70% of total).

We found that compared to placebo or reference therapy, treatment with GLP-1RAs was associated with a significant improvement in the absolute percentage of liver fat content, as assessed by magnetic resonance-based techniques (*n* = 7 RCTs; pooled WMD: −3.92%, 95% CI −6.27% to −1.56%, *p* < 0.0001), as well as in serum liver enzymes (mainly serum ALT and GGT levels). Notably, the results of our meta-analysis also show for the first time that among patients with biopsy-proven NASH and fibrosis, a significantly higher percentage of patients had resolution of NASH without worsening of liver fibrosis with once-daily subcutaneous treatment with either liraglutide or semaglutide than with placebo (*n* = 2 RCTs; pooled random-effects odds ratio 4.06, 95% CI 2.52–6.55; *p* < 0.0001). Conversely, there was no significant difference in the percentage of patients with an improvement in fibrosis stage without worsening of NASH in those treated with either liraglutide or semaglutide compared to those treated with placebo (pooled random-effects odds ratio 1.50, 95% CI 0.98–2.28; *p* = 0.06). It is possible that these two placebo-controlled RCTs were not of sufficient duration for improvements in liver fibrosis stage to become apparent, especially since most of the included patients had advanced fibrosis. In this meta-analysis we also found that compared to placebo or reference therapy, treatment with GLP-1 RAs was associated with significant reductions in body weight (~4 kg) and hemoglobin A1c levels (~0.5%). Importantly, all RCTs reported that treatment with GLP-1 RAs was well tolerated with a rate of adverse events not exceeding that of either placebo or reference therapy, except for a greater frequency of transient, mild-to-moderate gastrointestinal disorders.

Looking at the RCTs included in our meta-analysis, it clearly emerges that there is currently a dearth of large, high-quality RCTs with a sufficiently long duration and liver biopsy data, which is the “gold standard” method for assessing the drug-induced resolution of NASH or improvement in liver fibrosis stage. Indeed, most of the eligible RCTs have a small sample size (most RCTs included nearly 25–30 individuals for each arm of treatment) and a relatively short period of treatment (i.e., a median period of 26 weeks with only two RCTs with a treatment duration of 48 weeks or more). Most importantly, to date, there are only two RCTs testing the effects of once-daily subcutaneous treatment with liraglutide or semaglutide on resolution of NASH and/or improvement in liver fibrosis stage, which are the two histological features of NAFLD most strongly associated with risk of adverse liver-related and extra-hepatic outcomes in people with NAFLD [10,42,43,44]. Conversely, there is now a large body of evidence showing that treatment with liraglutide or other long-acting GLP-1 RAs exerts beneficial effects on cardiovascular, mortality, and kidney outcomes in people with T2DM [45,46,47,48,49]. In a systematic review and meta-analysis of seven large cardiovascular outcome trials (involving a total of ~56,000 participants), Kristensen et al. [50] showed that GLP-1 RAs significantly reduced major adverse cardiovascular events by 12%, all-cause mortality by 12%, hospital admission for heart failure by ~10%, and chronic kidney disease by 17% in individuals with T2DM. Collectively, these findings represent an attractive bonus for the long-term use of GLP-1 RAs in people with T2DM and NAFLD [20].

An in-depth analysis of the putative biological mechanisms through which GLP-1 RAs may exert their beneficial effects on NAFLD is beyond the scope of the present meta-analysis. However, it is reasonable to assume that the beneficial effects of liraglutide and other GLP-1 RAs on the individual histologic scores of NASH are multifactorial and a consequence of their combined effects on hyperglycemia/insulin resistance, weight loss and a direct beneficial effect on the liver (beyond the reduction in body weight and hyperglycemia). In fact, GLP-1 RAs are efficacious for treatment of T2DM and are also able to promote significant weight loss (on average 4–5 kg) [51]. Experimental evidence based on both human hepatocytes and animal models also suggests that GLP-1 RAs are able to improve hepatic steatosis by reducing *de novo* lipogenesis, enhancing oxidation of fatty acids and improving multiple elements of the insulin signaling pathways [52,53,54,55,56]. Moreover, preclinical NASH studies also suggested that GLP-1 RAs may reduce hepatic inflammation through mechanisms that are at least in part independent of body weight reduction [57].

The major strength of our study lies in the use of a systematic review methodology to identify all relevant RCTs (published up to 15 December 2020) that meet predefined inclusion criteria. This is the largest and most updated assessment to date on the efficacy of GLP-1 RAs to specifically treat NAFLD or NASH that has also included the most recently published RCTs using two long-acting GLP-1 RAs, such as dulaglutide and semaglutide [33,34]. In addition, most of the included RCTs have used magnetic resonance-based techniques, which have been shown to accurately quantify changes in liver fat content [58]. Finally, our meta-analysis suggests a possible beneficial class effect of GLP-1 RAs on surrogate indices of NAFLD, such as serum liver enzyme levels and imaging-detected liver fat content. We pinpoint that the current lack of any formal head-to-head RCTs prevents us from confidently ascertaining which of the four GLP-1 RAs tested is the most effective on NAFLD or NASH. However, it should be noted that liraglutide and semaglutide are the only two GLP-1 RAs, which have also been demonstrated to exert beneficial effects on NASH resolution so far. We acknowledge some important limitations of this meta-analysis that are strictly inherent to the RCTs included. First, as previously mentioned, most of the eligible RCTs have a relatively small sample size and a relatively short duration of treatment (i.e., a median period of 26 weeks). Second, only two RCTs with liver histological endpoints as a primary outcome were available for the meta-analysis. Although MRI-PDFF and MRS can provide accurate information regarding the changes in liver fat content, their accuracy for detecting the presence of NASH and assessing the stage of liver fibrosis is somewhat limited. Third, in the RCT with semaglutide all participants were randomly assigned to receive once-daily semaglutide at a dose of 0.1 mg, 0.2 mg, 0.4 mg/day or placebo [34]. However, the approved dosages of semaglutide for treatment of T2DM are 0.5 mg or 1.0 mg once weekly. Therefore, it remains currently uncertain which is the applicability and transferability of the RCT’s findings to clinical practice. Fourth, most of the eligible RCTs included individuals with NAFLD and T2DM (~70% of total participants), implying that larger RCTs in non-diabetic individuals with NAFLD are urgently awaited. Importantly, given that sex-related differences in the prevalence, risk factors and clinical outcomes of NAFLD are a distinctive and recently identified feature of NAFLD and NASH [59,60], future adequately powered RCTs should be specifically designed to explore sex differences in the response rate of NAFLD and NASH to treatment with GLP-1 RAs. Finally, analysis of changes of relevant metabolites in individuals with NAFLD or NASH, who are treated with GLP-1 RAs, also remains a research priority.

## 4. Materials and Methods

### 4.1. Registration of Review Protocol

The protocol for this meta-analysis was registered in advance on Open Science Framework registries (no: osf.io/tq87p).

### 4.2. Search Strategy and Study Selection

We performed a systematic review and meta-analysis of RCTs in accordance with the Preferred Reporting Items for Systematic Reviews and Meta-Analyses (http://www.prisma-statement.org) [61]. Eligible studies were identified by systematically searching PubMed, Scopus and ClinicalTrials.Gov databases from the inception date to 15 December 2020 (date of the last research), using the following free text terms: “nonalcoholic fatty liver disease” (OR “NAFLD” OR “nonalcoholic steatohepatitis” OR “NASH”) AND “glucagon-like peptide-1 receptor agonists” OR “GLP-1 receptor agonists” OR “exenatide” OR “liraglutide” OR “lixisenatide” OR “albiglutide” OR “dulaglutide” OR “semaglutide”. Eligible searches were limited to placebo-controlled or active-controlled RCTs involving adult individuals with NAFLD (regardless of diabetes status), in which the diagnosis of NAFLD was based on liver biopsy or imaging techniques, such as ultrasonography, computed tomography, and magnetic resonance-based techniques, such as MRI-PDFF or MRS. Reference lists of relevant papers and previous review articles were hand searched for other relevant studies. Studies enrolling individuals with significant alcohol consumption (usually defined as alcohol consumption > 30 g/day for men and >20 g/day for women, respectively) or secondary causes of chronic liver disease were excluded. Moreover, non-English-language articles and studies reported only in conference abstracts, unpublished studies, retrospective observational studies or non-randomized interventional studies were excluded. Two investigators (A.M. and G.T.) independently screened citations and assessed the excluded citations. These two investigators independently evaluated full-text articles by applying the inclusion criteria and resolved disagreements by consensus.

### 4.3. Data Extraction and Quality Assessment

The following data from the eligible RCTs were extracted: main study characteristics, sample size, length of the trial, type of intervention and dosages of GLP-1 RAs or active drug comparators, methods used for diagnosing NAFLD, as well as results for effectiveness and harms outcomes. Specifically, the primary outcome measures of interest were changes in the absolute percentage of liver fat content on imaging techniques and in serum liver enzyme levels, as well as in the percentage of histological resolution of NASH with no worsening of liver fibrosis and/or improvement in liver fibrosis stage without worsening of NASH. As secondary outcome measures of interest, we extracted data on changes in body weight and hemoglobin A1c levels and, whenever available, information on percentage of withdrawals due to severe adverse events. We did not contact any corresponding authors of the included RCTs in order to obtain additional information for the meta-analysis.

Two investigators (A.M. and G.T.) independently evaluated the risk of bias for each eligible RCT. For this purpose, we used the Cochrane Collaboration’s tool, which evaluates seven potential sources of bias: random sequence generation (selection bias), allocation concealment (selection bias), blinding of participants and personnel (performance bias), blinding of outcome assessment (detection bias), incomplete outcome data (attrition bias), selective reporting (reporting bias), and other bias [62]. For each of these domains, we categorized each eligible RCT into three categories: low, unclear, or high risk of bias [62].

### 4.4. Data Synthesis and Analysis

The effect sizes of the primary outcome measures of interest between patients randomly assigned to the placebo/reference therapy or those randomly assigned to treatment with GLP-1 RAs for each RCT were displayed either as weighted mean difference (WMD) and 95% confidence intervals (CI) for the changes in imaging-defined liver fat content and serum liver enzyme levels, or as the odds ratios (OR) and 95% CIs for the resolution of NASH without worsening of liver fibrosis and/or improvement in liver fibrosis stage with no worsening of NASH. The overall estimate of the effect size was computed using a random-effects model (i.e., DerSimonian-Laird model) [62]. If the outcome measures of interest were reported in median, range, or 25th–75th percentiles, the mean and SD values were estimated using validated formulas [63]. In addition, for continuous variables, if not available, SDs of the mean differences were estimated using the following formula: SD = [(SD pre-treatment)^2^ + (SD post-treatment)^2^ − (2R × SD pre-treatment × SD post-treatment)]${}^{\frac{1}{2}}$ [64]. Given that the pretest-posttest correlation coefficients (R) were not reported in the eligible RCTs, an R value of 0.5 was assumed in this meta-analysis [64].

Visual inspection of the forest plots was used to estimate the heterogeneity. The heterogeneity among the included RCTs was also tested by the I^2^-statistics. Specifically, the interpretation of the I^2^-statistics is as follows: I^2^-values of roughly 25% show low heterogeneity, I^2^-values of roughly 50% show medium heterogeneity, whereas I^2^-values of roughly 75% show high heterogeneity [65]. Publication bias was assessed by both the visual inspection of the funnel plots and the Egger’s regression test [66].

All statistical tests were two sided and used a significance level of *p*-value < 0.05. All statistical analyses were performed using the software STATA^®^ 16.1 with the meta-analysis package (STATA, College Station, TX, USA).

## 5. Conclusions

In conclusion, our meta-analysis is the most comprehensive and updated assessment of placebo-controlled and active-controlled RCTs of individuals with NAFLD, using different GLP-1 RAs to specifically treat NAFLD or NASH. Our meta-analysis supports the efficacy of GLP-1 RAs (especially liraglutide and semaglutide) in improving NAFLD, as assessed by magnetic resonance-based techniques or liver histology. This was true also for the two small RCTs conducted in China that examined the changes in hepatic steatosis by using ultrasonography. However, it is important to remark that no robust data from large RCTs with liver histological endpoints are currently available to comment on the long-term efficacy of GLP-1 RAs as a treatment for NASH. That said, if these promising results will be confirmed in larger and longer phase-3 RCTs with liver histological endpoints, it is reasonable that GLP-1 RAs will become a suitable treatment option (alone or in combination with other pharmacotherapies) in people with NAFLD or NASH, especially in those who are obese or have T2DM.

## Data Availability

All data are available in the manuscript and in the online-only supplementary material.

## Supplementary Materials

The following are available online at https://www.mdpi.com/2218-1989/11/2/73/s1, Figure S1: The PRISMA flow diagram for search and selection processes of the meta-analysis, Figure S2: Forest plot of the effects of different GLP-1 RAs on body weight (panel A) and hemoglobin A1c levels (panel B) as compared with placebo or reference therapy. The effect size was expressed as weighted mean difference (WMD) and 95% confidence intervals for all RCTs included, Figure S3: Univariable meta-regression analyses. A meta-analysis of the association of age (panel A), body mass index (panel B), and percentage of male sex (panel C) with weighted mean difference (WMD) of liver fat content (for RCTs using magnetic resonance-based techniques), Figure S4: Funnel plots of standard errors by weighted mean difference (WMD) in liver fat content as assessed by MRI-PDFF or MRS (panel A), serum ALT (panel B), serum AST (panel C), and serum GGT (panel D) levels. *p*-values were assessed by the Egger’s regression test, Table S1: Placebo-controlled or active-controlled RCTs of different GLP-1 RAs for treatment of NAFLD or NASH (*n* = 11 studies ordered by publication year), Table S2: Risk of bias for each RCT assessed by the Cochrane Collaboration’s tool.
