# Supplementary material for: Glucagon-Like Peptide-1 Receptor Agonists for Treatment of Nonalcoholic Fatty Liver Disease and Nonalcoholic Steatohepatitis: An Updated Meta-Analysis of Randomized Controlled Trials
Source: Metabolites. 2021 Jan 27;11(2):73. doi: 10.3390/metabo11020073 (PMC7911747; doi:10.3390/metabo11020073)
Supplement: Supplementary file 1 [file metabolites-11-00073-s001.pdf]

Online-Only Supplementary Material

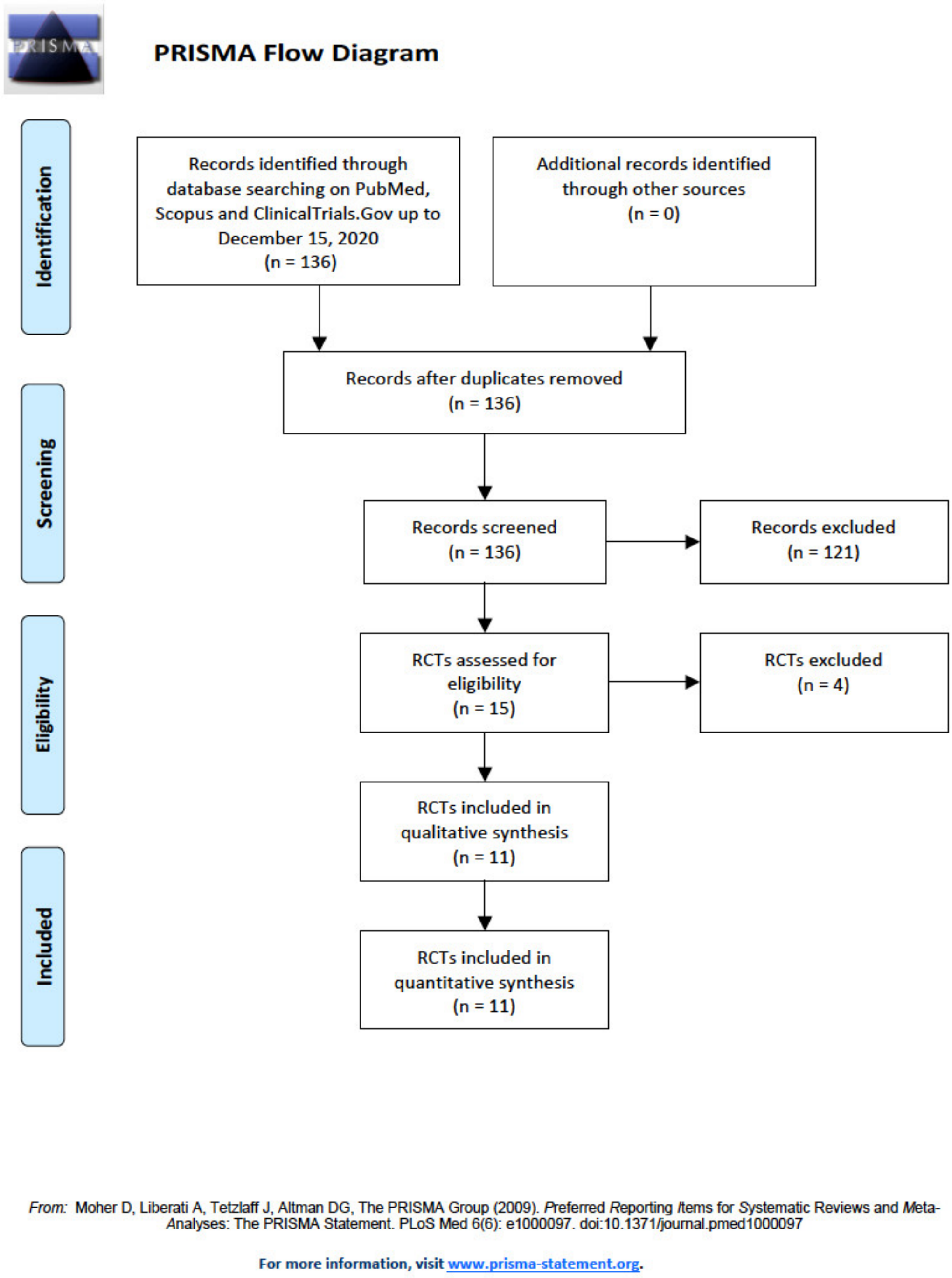

Figure S1. The PRISMA flow diagram for search and selection processes of the meta-analysis.

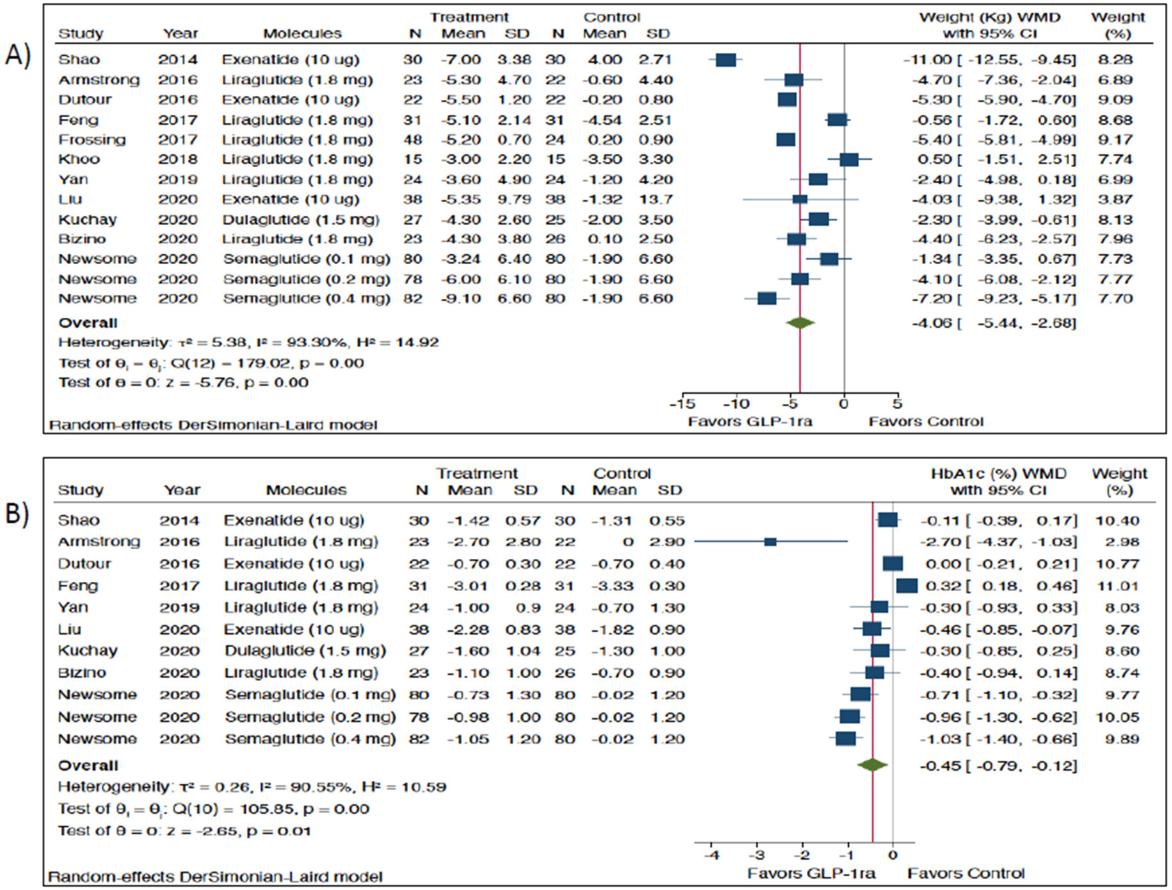

Figure S2. Forest plot of the effects of different GLP-1 RAs on body weight (panel **A**) and hemoglobin A1c levels (panel **B**) as compared with placebo or reference therapy. The effect size was expressed as weighted mean difference (WMD) and 95% confidence intervals for all RCTs included.

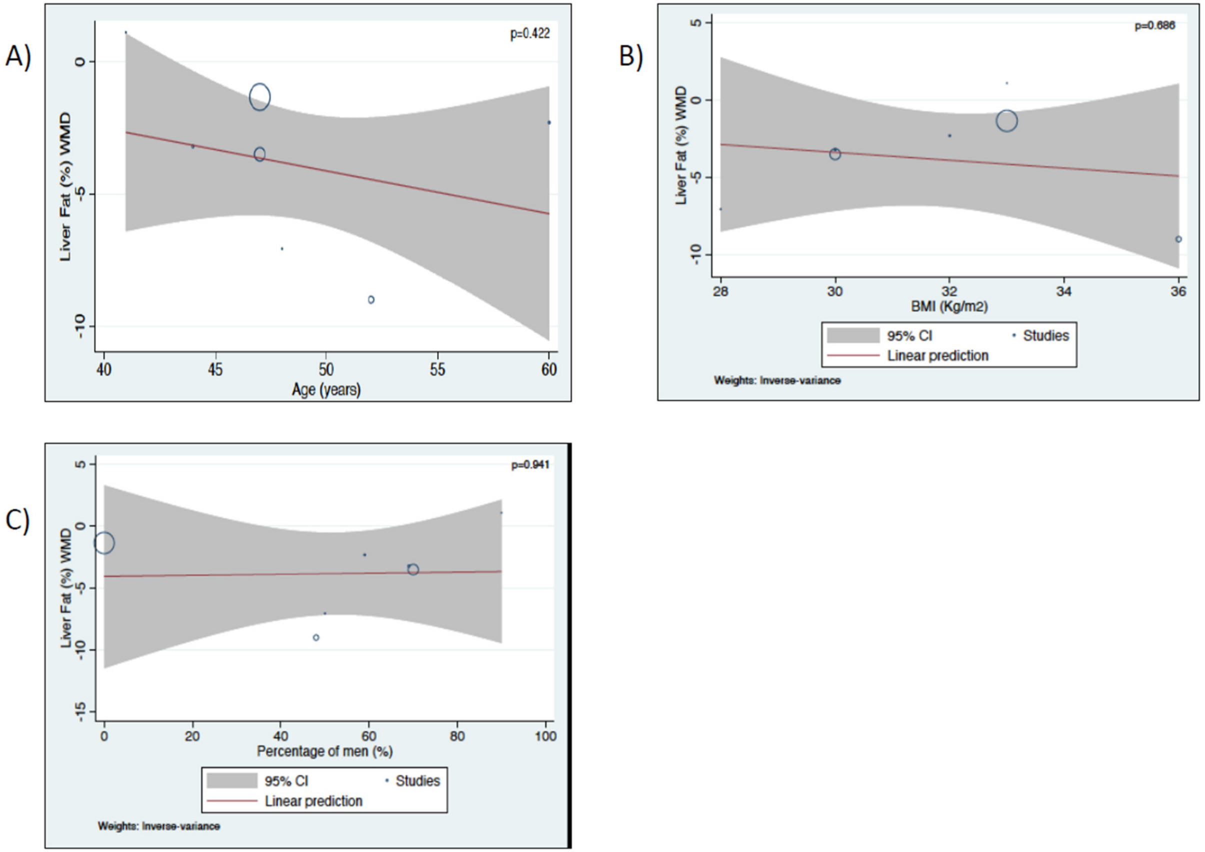

Figure S3: Univariable meta-regression analyses. A meta-analysis of the association of age (panel **A**), body mass index (panel **B**), and percentage of male sex (panel **C**) with weighted mean difference (WMD) of liver fat content (for RCTs using magnetic resonance-based techniques).

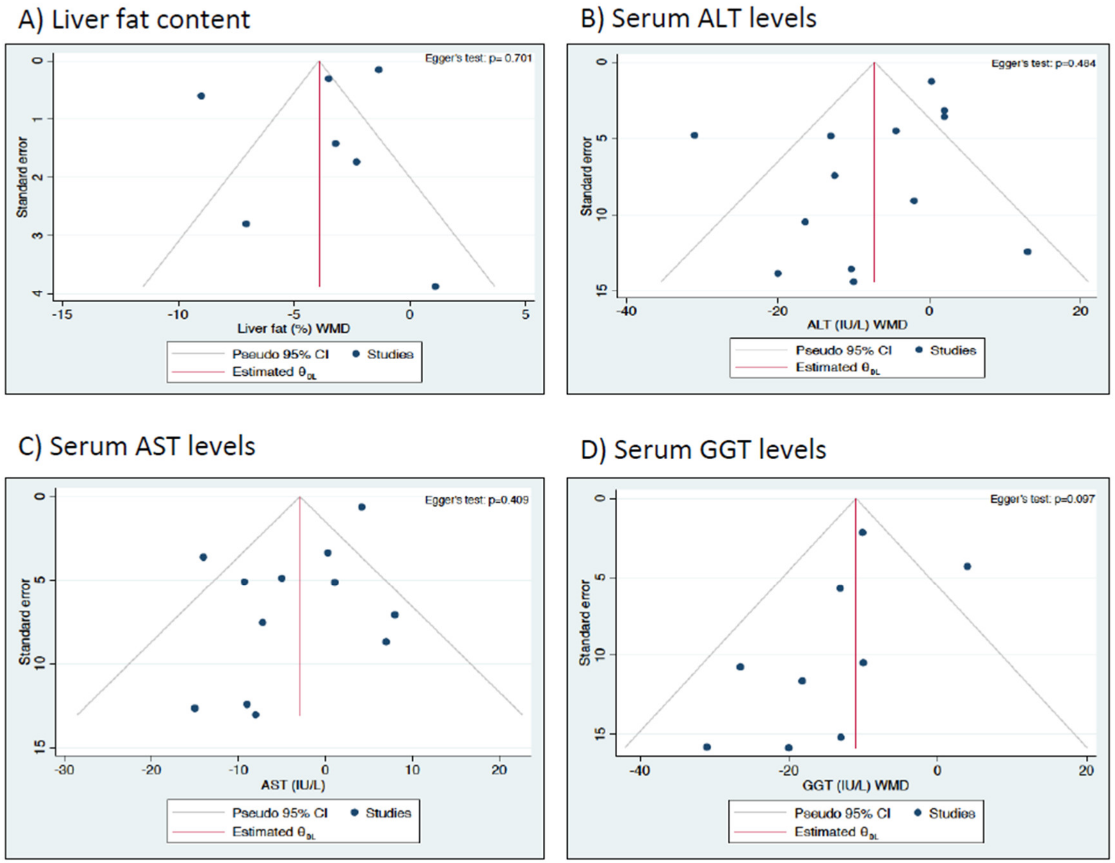

Figure S4: Funnel plots of standard errors by weighted mean difference (WMD) in liver fat content as assessed by MRI-PDFF or MRS (panel **A**), serum ALT (panel **B**) serum AST (panel **C**) and serum GGT (panel **D**) levels. P-values were assessed by the Egger's regression test.

Table S1. Placebo-controlled or active-controlled RCTs of different GLP-1 RAs for treatment of NAFLD or NASH (n = 11 studies ordered by publication year).

Author, Year, Country (PMID)	RCT's characteristics	Interventions (n), RCT's length	Efficacy and/or effectiveness outcomes A vs. B (or vs. C or D)	Major adverse effects
Shao *et al.* 2014; China (PMID: 24823873)	Patients with T2DM and NAFLD on liver ultrasound (with raised serum liver enzyme levels)<br>Mean age: 43 years; male sex: 48%; BMI 30 kg/m²; HbA1c 7.6%; ALT 166 IU/L; AST 123 IU/L	A. Exenatide + glargine (n = 30)<br>B. Intensive insulin: Insulin aspart + insulin glargine (n = 30)<br>Length: 12 weeks	Reversal rate of NAFLD based on ultrasound (A vs. B): 93% vs. 67%, p < 0.01<br>Differences in body weight change post-treatment *minus* pre-treatment: -7.8 vs. 3.3 kg, p < 0.001<br>No difference in HbA1c changes between groups	Not reported
Armstrong *et al*. 2016; United Kingdom (PMID: 26608256)	Patients with NASH (i.e., LEAN trial) on liver biopsy<br>Mean age: 51 years; male sex: 60%; BMI 36 kg/m²; ALT 71 IU/L; AST 51 IU/L; fibrosis F3-F4 (on histology) 52%; pre-existing T2DM: 33%	A. Liraglutide 1.8 mg/day (n = 26)<br>B. Placebo (n = 26)<br>Length: 48 weeks	Histologic resolution of NASH: 39% vs. 9%, p = 0.019<br>Change in histologic NAS score: −1.3 vs. −0.8, p = 0.24<br>Change in fibrosis stage: −0.2 vs. 0.2, p = 0.11<br>Fibrosis improvement: 26% vs. 14%, p = 0.46<br>Fibrosis worsening: 9% vs. 36%, p = 0.04<br>Change in ALT: −26.6 vs. −10.2 UI/L, p = 0.16<br>Change in AST: −27 vs.+9 IU/L; p = 0.02	Moderate gastro-intestinal disorders in the liraglutide vs. placebo: 81% vs. 65%
Dutour *et al*. 2016; France (PMID: 27106272)	Patients with T2DM, 95% of whom had NAFLD on MRS<br>Mean age: 52 years; male sex: 48%; BMI 36 kg/m2; HbA1c 7.5%; ALT 29 IU/L; AST 22 IU/L	A: Exenatide 5-10 mcg bid (n = 22)<br>B: Placebo (n = 22)<br>Length: 26 weeks	Exenatide and reference treatment led to a similar improvement in HbA1c (−0.7 ± 0.3% vs. −0.7 ± 0.4%; p = 0.29)<br>Significant weight loss was observed in the exenatide group (−5.5 ± 1.2 kg vs. −0.2 ± 0.8 kg; p = 0.001 for difference between groups)<br>Exenatide induced a significant reduction in liver fat content, compared with the reference treatment (liver fat content: −23.8 ± 9.5% vs. +12.5 ± 9.6%, p = 0.007)	Not reported

Feng *et al*. 2017; China (PMID: 28332301)	Patients with T2DM and NAFLD on ultrasound<br>Mean age: 47 years; male sex: 75%; BMI 28 kg/m^2; HbA1c 9.1%; ALT 49 IU/mL; AST 31 IU/L	A. Liraglutide up to 1.8 mg/d (*n* = 31)<br>B. Metformin up to 2000 mg/d (*n* = 31)<br>C. Gliclazide 60-120 mg/d (*n* = 31)<br>Length: 24 weeks	Liver fat content (estimated by ultrasound) decreased in all treatment groups, from 36.7 ± 3.6% to 13.1 ± 1.8% in the liraglutide group, from 33.0 ± 3.5% to 19.6 ± 2.1% in the gliclazide group, and from 35.1 ± 2.3% to 18.4 ± 2.2% in the metformin group (p<0.001 for all treatment groups, final vs. baseline)<br>Reduction in liver fat content following liraglutide treatment was greater than that following gliclazide treatment (*p* = 0.001)<br>Both liraglutide and metformin treatments reduced weight and improved liver function tests<br>HbA1c levels were lower in the liraglutide- and metformin-treated groups than in the gliclazide-treated group	Not reported
Frossing *et al*. 2018; Denmark (PMID: 28681988)	Non-diabetic women with polycystic ovary syndrome and NAFLD on MRS<br>Mean age: 47 years; female sex: 100%; BMI 33 kg/m^2	A. Liraglutide 1.8 mg/d (*n* = 48)<br>B. Placebo (*n* = 24)<br>Length: 26 weeks	Liraglutide treatment reduced body weight by 5.2 kg (-5.6% from baseline), liver fat content (on MR spectroscopy) by 44% and the prevalence of NAFLD by about two-thirds (all *p* < 0.01)<br>Liraglutide treatment caused significant reductions in fasting plasma glucose (liraglutide vs placebo, mean between-group difference [95% CI], −0.24 [−0.44 to −0.04] mmol/L; mean HbA1c [95% CI], −1.38 [−2.48 to −0.28] mmol/mol)	Nausea and constipation in the liraglutide group
Yan *et al*. 2019; China (PMID: 30341767)	Patients with T2DM and NAFLD on MRI-PDFF<br>Mean age: 44 years; male sex: 69%; BMI 29.8 kg/m^2; HbA1c 7.7%; ALT 43 IU/L; AST 33 IU/L	A. Liraglutide 1.8 mg/d (*n* = 24)<br>B. Insulin glargine 0.2 IU/kg/d (*n* = 24)<br>C. Sitagliptin 100 mg/d (*n* = 27)<br>Length: 26 weeks	In the liraglutide and sitagliptin groups, liver fat content, significantly decreased from baseline to week 26 (liraglutide, 15.4 ± 5.6% to 12.5 ± 6.4%, *p* < 0.001; and sitagliptin, 15.5 ± 5.6% to 11.7 ± 5.0%, *p* = 0.001)<br>HbA1c levels decreased in all treatment groups (liraglutide, 7.8 ± 1.4% to 6.8 ± 1.7%, *p* < 0.001; sitagliptin, 7.6 ± 0.9% to 6.6 ± 1.1%, *p* = 0.016; and insulin glargine, 7.7 ± 0.9% to 6.9% ± 1.1%, *p* = 0.013)<br>Body weight significantly decreased in the liraglutide and sitagliptin groups (but not in the insulin glargine group)	Not reported

Khoo *et al*. 2019; Singapore (PMID: 30721572)	Non-diabetic patients with obesity and NAFLD on MRI-PDFF<br>Mean age: 41 years; male sex: 90%; BMI 33 kg/m^2; ALT 88 IU/L; AST 48 IU/L	A. Liraglutide 3.0 mg/d (*n* = 15)<br>B. Lifestyle modifications (diet+exercise) (*n* = 15)<br>Length: 26 weeks	The two treatment groups had significant ($p < 0 .01$) and similar reductions in liver fat content (−8.1 ± 13.2 vs. −7.0 ± 7.1%), serum ALT (−39 ± 35 vs. −26 ± 33 U/L) and body weight at 26 weeks	Nausea, abdominal discomfort and diarrhoea in the liraglutide group
Liu *et al*. 2020; China (PMID: 31955491)	Patients with T2DM and NAFLD on MRI-PDFF<br>Mean age: 48 years; male sex: 50%; BMI 28 kg/m^2; HbA1c 8.3%; ALT 38 IU/L; AST 28 IU/L	A. Exenatide 1.8 mg/d (*n* = 38)<br>B. Insulin glargine 0.2 IU/kg/d (*n* = 38)<br>Length: 24 weeks	Liver fat content was significantly reduced after exenatide treatment (Δ liver fat −17.6 ± 12.9%). Exenatide treatment resulted in greater reductions in visceral adipose tissue decreased in the exenatide group compared to control group (ΔVAT −43.6 ± 68.2 cm^2), serum ALT, AST, GGT levels, BMI and waist circumference than control group	Proportion of adverse events were comparable between the two groups
Bizino *et al*. 2020; Netherlands (PMID: 31690988)	Patients with T2DM and NAFLD on MRS<br>Mean age: 60 years; male sex: 59%; BMI 32 kg/m^2; HbA1c 8.3%; ALT 14 IU/L; AST 33 IU/L	A. Liraglutide 1.8 mg/d (*n* = 23)<br>B. Placebo (*n* = 26)<br>Length: 26 weeks	Liver fat content was not different between groups (liraglutide 18.1 ± 11.2% to 12.0 ± 7.7%; placebo 18.4 ± 9.4% to 14.7 ± 10.0%; estimated treatment effect −2.1 [95% CI −5.3, 1.0]%)<br>Liraglutide vs. placebo significantly reduced body weight (liraglutide 98.4 ± 13.8 kg to 94.3 ± 14.9 kg; placebo 94.5 ± 13.1 kg to 93.9 ± 3.2 kg; estimated treatment effect −4.5 [95% CI −6.4, −2.6] kg)<br>Serum liver enzymes and HbA1c levels declined in both groups without a significant treatment effect of liraglutide vs. placebo (liraglutide HbA1c 8.4 ± 1.1% to 7.3 ± 1.2%]; placebo HbA1c 8.2 ± 1.0% to 7.5 ± 0.7%]	There were no serious drug-related adverse events

Kuchay *et al*. 2020; India (PMID: 32865597)	Patients with T2DM and NAFLD on MRI-PDFF (i.e. D-LIFT trial)<br>Mean age: 47 years; male sex: 70%; BMI 29.7 kg/m^2; HbA1c 8.4%; ALT 69 IU/L; AST 47 IU/L	A. Dulaglutide 1.5 mg/week (n = 32)<br>B. Placebo (n = 32)<br>Length: 24 weeks<br>Open-label trial (add-on to usual care)	Dulaglutide treatment resulted in a control-corrected absolute change in liver fat content of −3.5% (95% CI −6.6, −0.4; p = 0.025) and relative change of -26.4% (−44.2, −8.6; p = 0.004)<br>Dulaglutide-treated participants showed a significant reduction in serum GGT levels (mean between-group difference −13.1 U/l [95% CI −24.4, −1.8]; p = 0.025) and non-significant reductions in AST and ALT levels<br>Absolute changes in liver stiffness on Fibroscan (-1.31 kPa [-2.99, 0.37]; p = 0.12) were not significant when comparing the two groups.	There were no serious drug-related adverse events
Newsome *et al*. 2020; International cohort of individuals from 16 countries (PMID: 33185364)	Patients with NASH and fibrosis on liver biopsy<br>Mean age: 55 years; male sex: 41%; BMI 35.7 kg/m^2; pre-existing T2DM: 62% (HbA1c 7.3%); ALT 54 IU/L; AST 43 IU/L	A. Semaglutide 0.1 mg/day (n = 80)<br>B. Semaglutide 0.2 mg/day (n = 78)<br>C. Semaglutide 0.4 mg/day (n = 82)<br>D. Placebo (n = 80)<br>Length: 72 weeks	Percentage of patients in whom NASH resolution was achieved with no worsening of fibrosis was 40% in the 0.1-mg group, 36% in the 0.2-mg group, 59% in the 0.4-mg group, and 17% in the placebo group (p < 0.001 for semaglutide 0.4 mg vs. placebo)<br>Improvement in fibrosis stage occurred in 43% of the patients in the 0.4-mg group and in 33% of the patients in the placebo group (p = 0.48)<br>Treatment with semaglutide resulted in dose-dependent reductions of serum ALT and AST levels<br>Mean percent weight loss was 13% in the 0.4-mg group and 1% in the placebo group (p < 0.001)	Nausea, constipation, and vomiting were higher in the 0.4-mg group than in the placebo group

Abbreviations: ALT, alanine aminotransferase; AST, aspartate aminotransferase; BMI, body mass index; GGT, gamma-glutamyltransferase; MRS, magnetic resonance spectroscopy; MRI-PDFF, magnetic resonance imaging-proton density fat fraction; NAFLD, nonalcoholic fatty liver disease; NASH, nonalcoholic steatohepatitis; T2DM, type 2 diabetes mellitus.

Table S2. Risk of bias for each RCT assessed by the Cochrane Collaboration's tool.

Author(s)	Year	Random Sequence Generation (Selection Bias)	Allocation Concealment (Selection Bias)	Blinding of Participants and Personnel (Performance Bias)	Blinding of Outcome Assessment (Detection Bias)	Incomplete Outcome Data (Attrition Bias)	Selective Reporting (Reporting Bias)	Other Bias *
**Shao et al.**	2014	Low	Low	Unclear	Unclear	Unclear	Unclear	High
**Armstrong et al.**	2016	Low	Low	Low	Low	Low	Low	Low
**Dutour et al.**	2016	Low	Low	Low	Low	Unclear	Unclear	Unclear
**Feng et al.**	2017	Low	Unclear	Low	Low	Low	Unclear	High
**Frossing et al.**	2018	Low	Low	Low	Low	Unclear	Unclear	Unclear
**Yan et al.**	2019	Low	Low	Unclear	Low	Unclear	Unclear	Unclear
**Khoo et al.**	2019	Low	Low	Unclear	Low	Low	Unclear	Unclear
**Liu et al.**	2020	Low	Low	Unclear	Low	Unclear	Unclear	Unclear
**Bizino et al.**	2020	Low	Low	Low	Low	Low	Low	Unclear
**Kuchay et al.**	2020	Low	Low	Unclear	Low	Low	Low	Unclear
**Newsome et al.**	2020	Low	Low	Low	Low	Low	Low	Low

* Note: for each of the seven domains of the Cochrane Collaboration's tool the presence of low risk of bias was highlighted in green; unclear risk was highlighted in yellow, and high risk of bias was highlighted in red. Only two RCTs had paired liver biopsy data (i.e., the reference method for assessing drug-induced changes in hepatic steatosis, necro-inflammation or fibrosis), so we arbitrarily assigned an unclear risk of bias in the "Other Bias" domain of the Cochrane Collaboration's tool when RCTs used MRI-PDFF or MRS, or a high risk of bias when RCTs used liver ultrasound.
